# Phytochemical Screening, Antioxidant, and Antimicrobial Activities of Seven Underinvestigated Medicinal Plants against Microbial Pathogens

**DOI:** 10.1155/2022/1998808

**Published:** 2022-10-10

**Authors:** Borel Ndezo Bisso, Roland Njikang Epie Nkwelle, Roland Tchuenguem Tchuenteu, Jean Paul Dzoyem

**Affiliations:** ^1^Department of Biochemistry, Faculty of Science, University of Dschang, Dschang, Cameroon; ^2^Vally and You Commonwealth Institute for Natural Medicine (VACINAM) and Natural Medicine Research Laboratory, Bamenda, Cameroon

## Abstract

**Background:**

Plants are a rich source of therapeutic compounds that have tremendous applications in the pharmaceutical industry. This study aimed to identify the phytochemicals present in the seven selected medicinal plants as well as their antioxidant and antimicrobial activities.

**Methods:**

Phytochemical screening, total phenolic, and flavonoid contents were determined using standard methods. The antioxidant activity of plant extracts was determined using 2, 2-diphenyl-1-picrylhydrazyl (DPPH), hydroxyl (OH), and nitric oxide (NO) radical scavenging assays. The antimicrobial activity of the plant extracts was determined by the broth microdilution method.

**Results:**

The results of phytochemical analysis showed the presence of phenols, flavonoids, and steroids in all plant extracts. The extract of *Psychotria peduncularis* showed the highest total phenolic and flavonoid contents (5.57 ± 0.22 mg GAE/g and 1.38 ± 0.06 mg QE/g, respectively). All plant extracts showed very strong antioxidant activity against DPPH and NO radical scavenging with IC_50_ values ranging from 0.55 to 49.43 *µ*g/mL and 0.65 to 13.7 *µ*g/mL, respectively. The extracts of *Tristemma mauritianum* and *P. peduncularis* displayed significant antibacterial activity with MIC values ranging from 16 to 1024 *µ*g/mL. *T. mauritianum* extract showed bactericidal activity against all tested species. The extracts of *Alsophila manianna* and *P. peduncularis* showed significant antifungal activity (MIC = 64 *µ*g/mL) against *Candida albicans* strain.

**Conclusion:**

The screened extracts of medicinal plants used in our study can be used as potential antioxidant and antimicrobial agents, and resources for the development of new drugs.

## 1. Introduction

The emergence and spread of drug-resistant pathogens that have acquired new resistance mechanisms, leading to antimicrobial resistance, continues to threaten our ability to treat common infections [1]. Especially alarming is the rapid global spread of multi- and pan-resistant bacteria (also known as “superbugs”) that cause infections that are not treatable with existing antimicrobial medicines such as antibiotics or antifungals [2]. The clinical pipeline of new antimicrobials is dry. In 2019, the World Health Organization (WHO) identified 32 antibiotics in clinical development that address the WHO list of priority pathogens, of which only six were classified as innovative. Furthermore, a lack of access to quality antimicrobials remains a major issue. Antibiotic and antifungal shortages affect countries of all levels of development, especially in health-care systems [3].

In addition, the overproduction of reactive oxygen species (ROS) has been implicated in the development of various chronic and degenerative diseases such as cancer, respiratory, neurodegenerative, and digestive diseases [4]. Under physiological conditions, the concentrations of ROS are subtlety regulated by antioxidants, which can be either generated endogenously or externally supplemented. A combination of antioxidant-deficiency and malnutrition may render individuals more vulnerable to oxidative stress, thereby increasing the risk of cancer occurrence [4]. In addition, antioxidant defense can be overwhelmed during sustained inflammation such as in chronic obstructive pulmonary diseases, inflammatory bowel disease, neurodegenerative disorders, cardiovascular diseases, and aging [5]. Certain antioxidant vitamins, such as vitamin D, are essential in regulating biochemical pathways that lead to the proper functioning of organs. Antioxidant supplementation has been shown to attenuate endogenous antioxidant depletion thus alleviating associated oxidative damage in some clinical research [6]. Increasing trends of microbial resistance to antibiotics and various chronic and degenerative pathologies of humans caused by reactive oxygen species (ROS) have triggered the search for bioactive compounds from plants with alternative mechanisms of action to counteract pathogenic microbes and natural antioxidants capable of protecting the body against oxidative stress and free radical-induced damage [7, 8]. The proper use of medicinal plants requires accurate scientific information and an understanding of their chemical constituents. The therapeutic effects in plants are due to the chemical compounds therein [9]. Medicinal plants play a very important role in the development of alternative drugs without the adverse effects of synthetic drugs [10, 11]. Plants and natural products form the basis of both modern and traditional medicines and are currently widely used in the production of commercially produced drugs. Scientific and reliable reports indicated that about 25% of prescribed medicines worldwide are taken from herbs [12, 13].

*Heterotis decumbens*, *Lavigeria macrocarpa*, *Tristemma mauritianum*, *Cyanthillium stelluliferum*, *Alsophila manianna*, *Crassocephalum bougheyanum*, and *Psychotria peduncularis* are promising underinvestigated medicinal plants from Cameroon (Table 1). Although not indicated in the literature, they are used in Tombel locality in Cameroon for the treatment of microbial infections. *H. decumbens* of the Mecastomataceae family, it is largely used in traditional medicine for eye infection sprain, female infertility, trypanosomiasis, hernia, beriberi, and gastralgia [14]. *L. macrocarpa* is a traditional medicinal plant belonging to the Icacinaceae family and is used as a genital stimulant, depressant, and aphrodisiac [15]. *T. mauritianum* is a specie of flowering plants in the Mecastomataceae family. Previous studies on *T. mauritianum* reported its antioxidant and antisalmonellal activities [17]. Phytochemical investigation of *T. mauritianum* has resulted in the isolation of 2, 4-di-tert-butylphenol, 2 ((octyloxy) carbonyl) benzoic acid and sitosterol with antibacterial activity [18]. *C. stelluliferum,* also called *Triplotaxis stellulifera,* belongs to the Asteraceae family. Traditionally, it has been used for the treatment of polyhydramnios and amnionitis affecting newborns. It is also known to have immunomodulatory properties [19, 20]. *A. manianna* synomyn *Cyathae manianna* is a species of tree fern belonging to the Cyatheaceae family. Its leaves and seeds have been used to treat filariasis, while its stembark has been used for the treatment of backache [22, 23]. In addition, the antioxidant activity of *A. manianna* has been reported [24]. *C. bougheyanum* is a species of herb in the family Asteraceae. A previous study showed that *C. bougheyanum* did not produce any toxicity effect on Swiss albino mice [25]. *P. peduncularis* is a plant in the Rubiaceae family. It has been traditionally used in several countries to treat toothache, convulsion, yellow jaundice, stomachache, earache, backache, and skin infection [27].

Despite the traditional use of these medicinal plants, very little work has been done to investigate their phytochemical constituents. In addition, there are few studies on the antioxidant and antimicrobial activities of these medicinal plants. Therefore, in the present study, we evaluated the phytochemical constituents of extracts of these medicinal plants, and determined their antioxidant and antimicrobial activities against microbial pathogens.

## 2. Materials and Methods

### 2.1. Chemicals

DPPH (2, 2-diphenyl-1-picrylhydrazyl), (±)-*α*-tocopherol, Folin-Ciocalteu's reagent, dimethyl sulfoxide (DMSO), p-iodonitrotetrazolium chloride (INT), quercetin, gallic acid, ascorbic acid, butylated hydroxytoluene (BHT), ciprofloxacin, and ketoconazole were purchased from Sigma-Aldrich. The solvent and all reagents used in the analysis were of analytical grade.

### 2.2. Microorganisms and Media

Four fungal strains: *Candida albicans* (ATCC 90029), *Candida parapsilosis* (ATCC 22019), *Candida krusei* (ATCC 6258), and *Candida tropicalis* (ATCC 750) were used. The bacterial spp. used were *Escherichia coli* (ATCC 10536), *Staphylococcus aureus* (ATCC 25923), and *Enterobacter aerogenesis* (ATCC 13048), and three clinical isolates, namely, *Providencia stuartii*, *P. aeruginosa,* and *Vibrio cholerae* C06. Fungal and bacterial strains were obtained from the American Type Culture Collection (ATCC) while the clinical bacterial isolates were obtained from the Pasteur Institute Yaoundé (Cameroon). Mueller Hinton agar (MHA, Dominique Dutscher SAS) and Mueller Hinton broth (MHB, Dominique Dutscher SAS) were used for the activation of bacteria and antimicrobial assays, respectively. Sabouraud Dextrose agar (SDA, Liofilchem) and Sabouraud Dextrose broth (SDB, Liofilchem) were used for the activation of yeasts and antimicrobial assays, respectively.

### 2.3. Plant Sample Collection

Seven fresh plants (*H. decumbens*, *L. macrocarpa*, *T. mauritianum*, *C. stelluliferum*, *A. manianna*, *C. bougheyanum*, and *P. peduncularis*) (Table 1) were collected from various areas in the Tombel subdivision in southwest region of Cameroon in September 2016. The plants were authenticated at the Cameroon National Herbarium. The voucher number given for each plant is listed in Table 1.

### 2.4. Preparation of Plant Extracts

The collected plants were washed with water and dried in the shade at room temperature. Dried plant samples were powdered and 100 g of each plant sample powder was macerated with 800 mL of methanol. Then, each sample was filtered using Whatman No. 1 filter paper and from each filtrate the methanol was removed using a rotary evaporator (Buchi R-200) under reduced pressure. The extracts were stored at 4°C for further studies.

### 2.5. Preliminary Phytochemical Screening

The presence or absence of different constituents, such as alkaloids, steroids, glycosides, flavonoids, tannins, saponins, and terpenoids in each plant extract was determined using the method of Harbone (1984) [28]. Determination of the total phenolic content (TPC) and total flavonoid content (TFC) were performed using the method of Dzoyem and Eloff [29].

### 2.6. Antioxidant Assay

#### 2.6.1. DPPH Radical Scavenging Assay

The DPPH assay was performed using the method described by Dzoyem and Eloff [29]. Briefly, 900 *µ*L of DPPH solution (0.2 mM) prepared in methanol was mixed with 100 *μ*L of each plant extract sample at various concentrations (12.5 to 200 *μ*g/mL). After incubation in the dark at room temperature for 30 min, the absorbance of the mixture was measured at 517 nm using a spectrophotometer. Ascorbic acid was used as a positive control, methanol as a negative control, and extract without DPPH as a blank. The percent of inhibition of DPPH radical scavenging (%I) was calculated using the formula: %I = ((Absorbance_Control_ − Absorbance_Sample_)/Absorbance_Control_)) × 100. The concentration of each plant extract necessary to scavenge 50% of radicals (IC_50_) was calculated by plotting inhibition percentages against concentrations of each sample.

#### 2.6.2. Hydroxyl Radical Scavenging Assay

The hydroxyl radical scavenging assay of each plant extract was determined using the Fenton reaction as described by Sowndhararajan and Kang with slight modifications [30]. Briefly, 1.5 mL of each plant extract at different concentrations (12.5–200 *µ*g/mL) was mixed with 90 *µ*L of FeCl_3_ (4 mM) and 60 *µ*L of 1, 10-phenanthrolin (1 mM). Then, 2.4 mL of phosphate buffer saline (0.2 M pH 7.4) and 150 *µ*L of H_2_O_2_ (0.17 M) was added. The reaction mixture was incubated for 10 min at room temperature and the absorbance of the mixture was measured using a spectrophotometer at 560 nm. Buffer was used as a blank, and ascorbic acid was used as a positive control. The percent of inhibition of hydroxyl radical and IC_50_ were calculated as described above in the DPPH radical scavenging assay.

#### 2.6.3. Nitric Oxide Radical Scavenging Assay

The nitric oxide radical scavenging activity of each plant extract was measured as described by Kamble et al. with slight modifications [31]. The reaction mixture containing 0.75 mL of sodium nitropruisiate (10 mM), 0.5 mL of phosphate buffer (pH 7.4), and 0.5 mL of extract at different concentrations (12.5–200 *µ*g/mL) was incubated at room temperature for 1 h. Then, 1.2 mL of Griess reagent (1 mM) prepared in distilled water was added, and the reaction mixture was incubated at room temperature for 5 min. The absorbance was read using a spectrophotometer at 546 nm. The buffer was used as a blank, and ascorbic acid was used as a positive control. The percent of inhibition of nitric oxide radical and IC_50_ was calculated as described above in the DPPH radical scavenging assay.

### 2.7. Antimicrobial Activity

The minimum inhibitory concentration (MIC), minimum bactericidal concentration (MBC), and minimum fungicidal concentration (MFC) of the extracts were determined by the broth microdilution method [32]. Briefly, each plant extract (8192 *μ*g/mL) was serially diluted two-fold with MHB in a 96-well microplate at a total volume of 100 *µ*L. The concentrations of plant extracts ranged from 4096 to 2 *μ*g/mL. Then, wells were filled with 100 *µ*L of inoculum (1.5 × 10^6^ CFU/mL and 1.5 × 10^4^ CFU/mL for bacteria and yeast, respectively), and the microplate was incubated for 24 hours (bacteria) and 48 hours (yeast) at 37°C. Wells containing bacteria or fungi were used as the negative control while wells containing microorganisms and standard drugs (ciprofloxacin or ketoconazole) were used as the positive control. A volume of 40 *μ*L of INT solution (0.2 mg/mL) was added to each well and the microplate was incubated at 37°C for 30 min. Viable bacteria or yeast reduce the yellow dye of INT to a pink color. The MIC was recorded as the lowest extract concentration that prevented the color change in the medium. The MBC or MFC was determined by adding 50 *μ*L from the wells that did not show growth after incubation for the MIC test to 150 *μ*L of MHB (bacteria) or SDB (yeast). Then, the microplate was incubated at 37°C for 48 hours. MBC and MFC were defined as the lowest concentration of extract that killed all bacteria or yeast, respectively. The test was performed in triplicate and repeated three times.

The antibacterial activity of plant extracts was characterized as bactericidal (MBC/MIC ≤4) or bacteriostatic (MBC/MIC <4) [33]. Additionally, the antifungal activity of plant extracts was considered fungicidal when MFC/MIC ≤4 and fungistatic when MFC/MIC >4 [34].

## 3. Results

### 3.1. Phytochemical Analysis

The results of qualitative analysis of phytochemicals of the methanolic extracts of seven medicinal plants are shown in Table 2. It was observed that all plant extracts contained phenols, flavonoids, and steroids. The L. *macrocarpa* extract had all phytochemical constituents except anthraquinone. Additionally, saponins were present in all plants except *A. manniana* and *P. peduncularis.*

### 3.2. Total Phenolic and Flavonoid Contents

**Figure 1:**
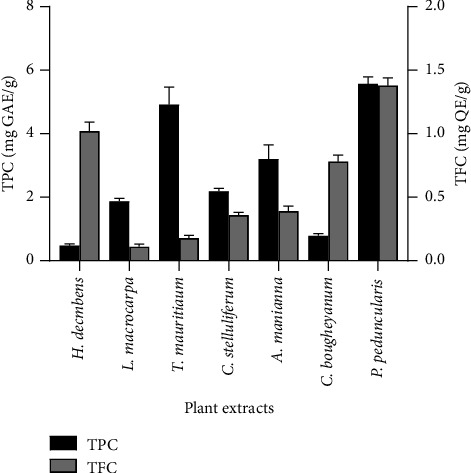
TPC and TFC of seven medicinal plant extracts.

The quantities of phenolic and flavonoid contents in the different medicinal plants are presented in Figure 1. The extracts of *P. penduncularis* and *T. mauritianum* presented the highest TPC (5.57 ± 0.22 mg GAE/g and 4.92 ± 0.55 mg GAE/g, respectively). However, the extracts of *C. bougheyanum* and *H. decumbens* presented the lowest TPC (0.79 ± 0.06 mg GAE/g and 0.48 ± 0.05 mg GAE/g, respectively). The plant extract of *P. peducularis* (1.38 ± 0.06 mg QE/g) presented the highest TFC while the plant extract of *L*. *macrocarpa* (0.11 ± 0.01 mg QE/g) showed the lowest TFC. The TFC of the *C. stelluliferum* (0.36 ± 0.02 mg QE/g) extract was similar to that of the *A. manniana* extract (0.39 ± 0.04 mg QE/g).

### 3.3. Antioxidant Activity

The antioxidant activities of medicinal plant extracts as determined by the DPPH, OH, and NO radical scavenging assays are shown in Table 3. The IC_50_ values of the plant extracts ranged from 0.55 to 49.43 *μ*g/mL and 0.65 to 13.7 *μ*g/mL in the DPPH and NO methods, respectively. Compared to ascorbic acid, the IC_50_ values of the *P. peduncularis* extract in the DPPH and NO methods were similar.

### 3.4. Antimicrobial Activity of Plant Methanolic Extracts

Table 4 shows the antimicrobial activity of seven medicinal plants against bacterial pathogens. The extracts of *T. mauritianum* and *P. peduncularis* showed the highest antibacterial activity with MIC values ranging from 16 to 1024 *µ*g/mL. Additionally, the *H. decumbens* extract showed important antibacterial activity with MIC values ranging from 32 to 1024 *µ*g/mL. However, the *L. macrocarpa* plant extract presented the lowest antibacterial activity (MIC values ≥ 2048 *µ*g/mL). *T. mauritianum* extract exhibited bactericidal activity against all tested species with an MBC/MIC ratio equal to 2. Ciprofloxacin was used as a control drug, and its MIC and MBC values ranged from 0.25 to 32 *µ*g/mL and 0.5 to 64 *µ*g/mL, respectively.

Concerning antifungal activity, the extract of *H. decumbens* displayed the best activity (MIC values ranging from 16 to 256 *µ*g/mL) followed by the extracts of *P. peduncularis* and *T. mauritianum* with MIC values ranging from 32 to 512 *µ*g/mL and 64 to 512 *µ*g/mL respectively. In addition, the extracts of *H. decumbens*, *T. mauritianum,* and *P. peduncularis* showed fungicidal activity against all fungal strains. However, the lowest antifungal activity was obtained for *L. macrocarpa,* with MIC values ranging from 256 to ≤2048 *µ*g/mL. Ketoconazole exhibited fungicidal activity against all tested fungal strains.

## 4. Discussion

The use of medicinal plants for their pharmacological properties is being increasingly reported in the different countries. The World Health Organization estimates that more than 25% of prescription drugs derived from plants [12, 35]. In the present study, the phytochemical analysis revealed the presence of phenols, flavonoids, and steroids in all extracts of medicinal plants. Due to their various biological properties, phenolic and flavonoid compounds are considered the most important classes of phytochemicals [36]. In fact, some effects of phenolic and flavonoid compounds include anti-inflammatory, antispasmodic, antiulcer, antidepressant, antidiabetic, cytotoxicity and antitumor, antimicrobial, and antioxidant properties. Additionally, steroids derived from medicinal plants are known to possess antibacterial and insecticidal properties [37]. These results are in agreement with those obtained by Ngbolua et al., who found that *A. manniana* contained flavonoids, quinones, tannins, terpenoids, and steroids [24]. In addition, similar funding was obtained by Wickens and Burkill, who showed the presence of tannins in the extract of *C. stelluliferum* [21]. Our results showed that saponins were present in all plants except *C. stelluliferum* and *P. peduncularis.* Plant extracts containing saponins have been used to treat inflammation, cerebrovascular and cardiovascular diseases, gastric ulcers, and ultraviolet damage [38]. In addition, saponins have been used as adjuvants to enhance the absorption of bioactive molecules and drugs [39]. The presence of these phytochemical compounds in the plant extracts of this study could be the reason for their use as a traditional medicine by the population of Tombel subdivision.

The total phenolic and flavonoid contents in selected medicinal plants were also investigated. The extracts of *P. penduncularis* presented the highest TPC and TFC. The high amounts of phenolic and flavonoid compounds in this plant could increase its biological properties compared to other studied medicinal plants. The antioxidant activity should not be concluded on the basis of a single method [40]. In order to determine the antioxidant activity of studied medicinal plants, DPPH, OH, and NO radical scavenging assays were used. Antioxidant activity is considered as follows: very strong (IC_50_ < 50 *µ*g/mL), strong (50 ≤ IC_50_ < 100 *µ*g/mL), moderate (100 ≤ IC_50_ < 150 *µ*g/mL), and low (IC_50_ > 150 *µ*g/mL) [41]. On this basis, all plant extracts showed very strong antioxidant activity DPPH and NO radical scavenging activity. Additionally, *C. stelluliferum* and *C. bougheyanum* extracts exhibited strong OH scavenging activity with IC_50_ values of 79.06 *µ*g/mL and 67.29 *µ*g/mL, respectively. This antioxidant activity observed in the studied medicinal plants could be attributed to the presence of phenolic compounds such as phenolic acids and flavonoids. These phenolic compounds act as antioxidants by hydrogen-donating properties of their phenolic group hydroxyls [42]. Additionally, phenolic compounds can chelate the metal ions involved in the production of ROS [43]. Our results are similar to those obtained by Ngbolua et al., who reported the antioxidant activity of *A. manniana* [24]. Additionally, Tsafack et al. reported the antioxidant activity of *T. mauritianum* [17].

Plants are a good source of new medicine. In our study, we also tested the antimicrobial activity of seven medicinal plants against bacterial and fungal pathogens. The antibacterial or antifungal activity is considered significant (MIC <100 *μ*g/mL), moderate (100 ≤ MIC ≤625 *µ*g/mL), and low (MIC >625 *µ*g/mL) [11]. On this basis, the *H. decumbens* extract showed significant antibacterial activity (MIC = 32 *µ*g/mL) against *P. stuartii* isolates. In addition, the extracts of *T. mauritianum* and *P. peduncularis* displayed significant antibacterial activity (MIC = 16 *µ*g/mL) against *S. aureus* strain. Concerning antifungal activity, the extracts *H. decumbens*, *T. mauritianum,* and *P. peduncularis* exhibited significant activity against *C. krusei* strain. Additionally, *A. manianna* and *P. peduncularis* showed significant antifungal activity (MIC = 64 *µ*g/mL) against *C. albicans* strain. However, the majority of plant extracts exhibited moderate antibacterial and antifungal activities. The different antimicrobial activities of plant extracts could be attributed to the presence of phytochemical compounds such as phenolics, flavonoids, alkaloids, tannins, saponins, steroids, and triterpenes, which have antimicrobial properties and cause damage of the cell membrane, leading to cell death through its disruption [9]. In addition, these phytochemical compounds can inhibit of cell wall formation, mitochondrial dysfunction, DNA replication, protein synthesis, biofilm formation, and efflux pumps [44–46]. Several studies have demonstrated that medicinal plants containing phenolics, flavonoids, alkaloids, tannins, saponins, steroids, and triterpenes have the antimicrobial potential as bactericidal, bacteriostatic, fungicidal, or fungistatic agents against microbial pathogens [47–49]. Limited information exists on the antibacterial activity of these medicinal plants. However, Tsafack et al. reported the antibacterial activity of *T. mauritianum* against *Salmonella* [17].

## 5. Conclusion

The results of this study revealed the antibacterial and antifungal potential of the studied medicinal plants against drug-resistant pathogens. Additionally, these medicinal plants could be used as a natural source of antioxidants. Further purification and isolation of the bioactive compounds in these plant extracts would provide possible identification of the mechanism of action and possible lead compounds for the development of new drugs.

## Figures and Tables

**Table 1:** Characteristics of the medicinal plants investigated in this study.

Scientific names (Family) voucher number	Part used	Traditional use	Previous pharmacological studies	Isolated phytochemical compounds
H. decumbens (Mecastomataceae) 18026/SRF.Cam	Leaves	Eye infection sprain, female infertility, trypanosomiasis, hernia, beriberi, and gastralgia [14]	Not reported	Not reported

*L. macrocarpa* (Icacinaceae) 179761/SRF.com	Fruit	Genital stimulants/depressants, aphrodisiac [15]	Not reported	Not reported

*T. mauritianum* (Mecastomataceae) 6995/SRF-Cam	Leaves	Wounds, cough, and premenstrual tension [16]	Antisalmonellal and antioxidant [17]	2,4-di-tert-butylphenol 2 ((octyloxy) carbonyl) benzoic acid and sitosterol [18]

*C. stelluliferum* (Asteraceae) 20495/HNC	Whole plant	Amnionitis affecting the newborn, polyhydramnios [19]	Immunomodulatory [20]	Tannins [21]

*A. manniana* (Cyatheaceae) 25694/HNC	Leaves, seeds, Stembark	Filariasis [22] Backache [23]	Antioxidant [24]	Flavonoids, quinones, tannins, terpenoids, and steroids [24]

*C. bougheyanum* (Asteraceae) 7635/HNC	Whole plant	Not reported	Acute and sub-chronic toxicity [25]	Not reported

*P. peduncularis* (Rubiaceae) 37630/HNC	Leaves	Heart conditions [26] toothache, convulsion, yellow jaundice, stomachache, earache, backache, and skin infection [27]	Not reported	Not reported

**Table 2:** Qualitative analysis of phytochemicals of the methanolic extracts of seven medicinal plants.

Phytochemical groups	Plant extracts						
	*Hd*	*Lm*	*Tm*	*Cs*	*Am*	*Cb*	*Pp*
Alkaloids	−	+	−	+	−	+	−
Phenols	+	+	+	+	+	+	+
Flavonoids	+	+	+	+	+	+	+
Saponins	+	+	+	+	−	+	−
Triterpenes	+	+	−	−	+	−	+
Steroids	+	+	+	+	+	+	+
Anthraquinone	−	−	+	−	+	−	−
Tannins	+	+	+	+	+	−	+

+: presence of phytochemicals, −: absence of phytochemicals, *Hd*: *H. decumbens, Lm*: *L. macrocarpa, Tm*: *T. mauritianum, Cs*: *C. stelluliferum, Am*: *A. manniana, Cb*: *C. bougheyanum, Pp*: *P. peduncularis.*

**Table 3:** IC_50_ (*μ*g/mL) values of seven medicinal plant extracts against DPPH, OH, and NO radical scavenging.

	IC_50_ (*μ*g/mL)		
	DPPH	OH	NO
*H. decumbens*	35.07 ± 0.55	123.59 ± 0.23	10.44 ± 0.36
*L. macrocarpa*	49.43 ± 0.06	˃1000	0.78 ± 0.00
*T. mauritianum*	25.88 ± 0.54	169.82 ± 0.30	13.7 ± 0.81
*C. stelluliferum*	58.88 ± 0.59	79.06 ± 0.80	5.15 ± 7.07
*A. manianna*	37.15 ± 0.86	153.46 ± 1.94	7.34 ± 0.13
*C. bougheyanum*	30.97 ± 0.10	67.29 ± 0.55	5.58 ± 0.06
*P. peduncularis*	0.55 ± 0.00	512.86 ± 0.93	0.60 ± 0.00
Ascorbic acid	0.45 ± 0.00	52.6 ± 0.35	0.52 ± 0.00

**Table 4:** Minimum inhibitory concentration (MIC in *µ*g/mL), minimum bactericidal or fungicidal concentration (MBC or MFC in *µ*g/mL), and MBC or MFC/MIC ratio of the seven selected medicinal plants.

		Microorganisms									
		*Ec*	*Sa*	*Ps*	*Ea*	*Pa*	Vc06	*Ca*	*Ct*	*Cp*	*Ck*
*H. decumbens*	MIC	1024	128	32	256	—	128	256	128	256	16
	MBC	—	256	64	512	—	256	1024	512	512	64
	MBC/MIC or MFC/MIC	—	2	2	2	—	2	4	2	2	4

*L. macrocarpa*	MIC	2048	—	—	—	—	1024	256	1024	—	1024
	MBC	—	—	—	—	—	2048	1024	—	—	—
	MBC/MIC or MFC/MIC	—	—	—	—	—	2	4	—	—	—

*T. mauritianum*	MIC	128	128	512	16	256	64	256	128	512	64
	MBC	256	256	1024	32	512	256	512	512	1024	256
	MBC/MIC or MFC/MIC	2	2	2	2	2	4	2	4	2	4

*C. stelluliferum*	MIC	128	512	32	1024	—	512	128	512	1024	—
	MBC	256	1024	128	—	—	512	512	—	—	—
	MBC/MIC or MFC/MIC	2	2	4	—	—	1	4	—	—	—

*A. manianna*	MIC	256	1024	2048	—	—	2048	64	512	512	—
	MBC	1024	2048	—	—	—	—	256	1024	1024	—
	MBC/MIC or MFC/MIC	4	2	—	—	—	—	4	4	4	—

*C. bougheyanum*	MIC	256	512	1024	256	—	64	128	512	256	256
	MBC	512	512	2048	512	—	128	512	—	256	1024
	MBC/MIC or MFC/MIC	2	1	2	2	—		4	—	1	4

*P. peduncularis*	MIC	128	1024	128	16	1024	128	64	512	128	32
	MBC	512	—	512	32	2048	256	128	512	512	128
	MBC/MIC or MFC/MIC	2	—	2	2	2	2	2	1	2	4

Ciprofloxacin	MIC	0.25	0.5	1	0.5	0.5	1	Nd	Nd	Nd	Nd
	MBC	0.5	1	2	1	1	2	Nd	Nd	Nd	Nd
	MBC/MIC or MFC/MIC	2	2	2	2	2	2	Nd	Nd	Nd	Nd

Ketoconazole	MIC	Nd	Nd	Nd	Nd	Nd	Nd	4	8	2	4
	MBC	Nd	Nd	Nd	Nd	Nd	Nd	8	16	8	4
	MBC/MIC or MFC/MIC	Nd	Nd	Nd	Nd	Nd	Nd	2	2	4	1

MIC: minimum inhibitory concentration, MBC: minimum bactericidal concentration, MFC: minimum fungicidal concentration, *Ec*: *E. coli*, *Sa*: *S. aureus*, *Ps*: *P. stuartii*, *Ea*: *E. aerogenesis*, *Pa*: *P. aeruginosa*, *Vc*: *V. cholerae* C06, *Ca*: *C. albicans*, *Ct*: *C. tropicalis*, *Cp*: *C. parapsilosis*, *Ck*: *C. krusei*, —: >2048 *μ*g/mL, *Nd*: not determined.

## Data Availability

The data used to support the findings of this study are available upon reasonable request from the corresponding author.
